# Simulated Learning for Real-World Skills: Evaluating the Impact of Pediatric Informed Consent Training on Learner Communication in a Simulated Emergency Room

**DOI:** 10.7759/cureus.80167

**Published:** 2025-03-06

**Authors:** Shivani Dixit, Sarah Boulos, Sanlyn Buxner, David Eckhardt

**Affiliations:** 1 Simulation in Medical Education, Rocky Vista University, Parker, USA; 2 Simulation, Rocky Vista University College of Osteopathic Medicine, Parker, USA; 3 Statistics, University of Arizona, Tuscon, USA

**Keywords:** graduate medical education, informed consent, pediatrics, simulation, structured didactics

## Abstract

Informed consent is an integral part of medical care and can be especially complicated in the care of the pediatric population where adult caregivers provide consent. Despite the complexity and importance required in obtaining parental consent, many healthcare trainees (students and residents) do not feel comfortable securing consent. Obtaining informed consent is often not a standard part of the medical education curriculum, which adds to this level of discomfort. This study measured communication outcomes during obtaining informed consent during a simulated ER setting for 200 medical professional students when given a structured didactic curriculum. Results showed that in comparing the groups of those who received the intervention after obtaining consent with those who obtained the intervention before the consent, an independent t-test revealed a statistically significant difference in scores between the groups who did not complete the education module before completing the simulation (n=42, mean = 0.627, s.d. = 0.127) and the groups who did receive the education model before completing the simulation (n=42, mean = 0.685, s.d. = 0.136), *t*(82) = 2.023, p = 0.023, with a small effect size. The results of this study show that incorporating structured and formal didactic teaching modality about obtaining parental consent for pediatric populations can lead to better communication outcomes.

## Introduction

Informed consent is an important aspect of medical care and has special nuances when managing a pediatric population. Pediatric providers are challenged with the complex ethics of medical decision-making regarding consent, capacity, and surrogate decision-making [1]. While the integration of parental consent and pediatric assent is the standard of care by the American Academy of Pediatrics [2], there is still no widespread understanding or teaching of informed consent at the level of medical trainees [3]. While some formal assessments on informed consent at the medical school level have been conducted and have showcased the importance of this skill [4], formal teaching of obtaining parental consent for medical decision-making in pediatrics has not been well developed. Pediatric residents have often reported anxiety and discomfort associated with obtaining consent due to their lack of preparation and formative training [5].

While it is commonly accepted that improved physician-patient communication results in more positive patient outcomes [6], studies have shown that pediatric residents do not view informed consent as a critical component of this physician-patient relationship without formal didactic training [7]. While the issue of obtaining parental consent becomes most relevant during pediatric residency, the lack of didactic training begins at the medical school level. Despite the importance of obtaining proper informed consent, many medical students are not confident or competent in their ability to obtain informed consent [8].

Some pediatric residencies have studied the efficacy of shared decision-making after integrating structured didactic lessons on pediatric informed consent and obtaining parental consent, which has suggested more positive outcomes and better patient-physician relationships [7]. Some previous studies have designed smartphone applications as a method to incorporate the elements of informed consent to improve confidence for pediatric residents [9]; however, such applications are not widely used. Additionally, studies have determined key elements of parent satisfaction toward informed consent as giving parents more time to make their decision, the amount and type of information provided, organization of the consent conference, communication style, and providing additional materials [10]. While elements of structured didactic in obtaining informed consent have been highlighted, the efficacy of incorporating this education in preclinical medical education has not been effectively outlined.

While the role of didactic education models has not been well studied, the effects of the deliberate-practice model have been well established in clinical medical education [11]. These studies have suggested that deliberate practice is more effective than traditional teaching methods in acquiring a specific clinical skill. Studies have shown the efficacy of deliberate practice in acquiring nonclinical skills such as communication, empathy, and self-efficacy as well as in graduate medical education [12]. However, the exact effect of simulation-based deliberate practice on acquiring parental consent for pediatric patients is lacking in evidence. As deliberate practice is a proven teaching tool, this study proposed to compare a didactic teaching model to deliberate practice in communication outcomes.

This study aims to investigate the inclusion of didactic information on obtaining pediatric consent and parental consent in simulated patient encounters to improve outcomes. As the “see one-do one-teach one” approach is no longer effective in regard to informed consent [13], this study aimed to assess communication outcomes based on a simulated emergency department environment where both interprofessional and provider-patient communication was utilized. The study was conducted to answer the following research questions to evaluate quantitative communication outcomes during simulated pediatric cases of preclinical healthcare profession students when integrating structured pediatric consent didactic instruction during a simulated busy emergency department scenario. 

1. To what extent does incorporating formal didactic materials for obtaining telephone parental consent for pediatric patients affect how well students obtained consent? 

2. What is the effect of deliberate practice on outcomes? 

3. When students are taught using the classic deliberate practice model (those who received the didactic training before the first simulation), does deliberate practice prove to be a more powerful teaching tool; or is it more beneficial for students to have a clinical introduction before receiving the intervention?

## Materials and methods

First year preclinical Doctor of Osteopathy (DO) and Physician Assistant (PA) students from our university were enrolled in the interprofessional education (IPE) course. Students were participating in the emergency department-based simulation in this randomized-controlled study. The simulations took place over three days to accommodate all of the students. Groups of five to seven students, which were assigned at the beginning of the academic year, worked together to treat 14 standardized patients with a diverse range of common pathologies presented during a busy emergency department simulation scenario. On the day of the simulation exercise, the teams were made up of PA and DO students from our university and paramedic students from a local community college.

The educational intervention consisted of a structured information pamphlet that outlined the necessary communication and content topics for conducting pediatric informed consent and parental consent. This pamphlet also outlined scenarios in which parental consent is appropriate and when pediatric patients can consent to their own treatment. Students also watched a short video explaining the pamphlet. A video was used to standardize the teaching for all students. The components of the aforementioned educational model, including the written materials and the video instruction, are henceforth referred to as the intervention. In addition to the intervention, the authors looked at the effects of the well-established model of deliberate practice in simulation when analyzing the data.

Students received a pre-briefing instructional video that outlined the objectives and workflow of the event for that day. This ensured that students were prepared for the objectives and assessment of the simulation event. Each group of students was presented with seven pediatric cases that required appropriate parental and pediatric consent. If parental consent prior to treatment or a procedure was required, the students were instructed to call the patient’s “parent”, played by a designated faculty member who was assigned this role for the event. After completion of the simulation once (attempt 1), each group participated in a structured debrief with course faculty. After the debrief, students participated in the simulation a second time (attempt 2) as part of the deliberate practice model.

Student groups were sorted into three cohorts, randomized by which of the three days they were assigned to complete the event. On day 1, students received the intervention after they had completed both simulations, serving as the control. On day 2, students received the intervention in between the first and second simulations, during the instructor-led debrief. On day 3, students received the intervention at the beginning, prior to completing either simulation. To maintain the integrity of the intervention, no additional information to what was already highlighted in the pamphlet and associated instructional video was provided by faculty before, during, or after the simulation event.

The designated faculty member assigned to the “parent” role during the event used a standardized checklist based on the educational information presented to the students during the intervention and scored each team using a rubric (see Appendix). Checklist was in the form of a yes or no question indicating that the students either did meet the communication criteria or they did not. Data collected was then transferred into numerical form onto an Excel spreadsheet (Microsoft Corp., Redmond, WA) where all “yes” answers were documented as 1 and all “no” answers were documented as 0. The maximum possible score was a 9, which would equate to the student hitting all communication points related to obtaining proper telephone informed consent for pediatric cases.

## Results

For all research questions, analyses included inspecting the number of groups who tried to obtain consent for each attempt, descriptive statistics for each attempt including the average score and standard deviation of all groups for each attempt, and independent t-tests to compare scores from groups at different times. In comparing the groups of those who received the intervention after obtaining consent with those who obtained the intervention before the consent, a Levene’s test of equality of variances showed that the assumption of equality of variances was met between groups.

Data analysis by research question

Research Question 1

To answer this research question, group level outcome data was compared for those groups who attempted to obtain consent before receiving the intervention to those who received the intervention before attempting to obtain consent. This analysis compared all groups on day 1 and those who did attempt 1 on day 2 to those groups who did attempt 2 on day 2 and all groups on day 3. The analysis included descriptive statistics of the number of groups who attempted to obtain consent, means and standard deviations for each condition (intervention after or before) and an independent t-test to compare outcomes scores of these conditions. In comparing the groups of those who received the intervention after obtaining consent with those who obtained the intervention before the consent, an Independent t-test revealed a statistically significant difference in scores between the groups who did not complete the education module before completing the simulation (n=42, mean = 0.627, s.d. = 0.127) and the groups who did receive the education model before completing the simulation (n=42, mean = 0.685, s.d. = 0.136), *t*(82) = 2.023, p = 0.023, with a small effect size. This shows that overall groups, who received the intervention, received a better score on their second attempt to obtain consent (Table 1).

**Table 1:** Means of communication outcomes between those who received the intervention prior to completing simulation and those who received intervention after completion of simulation.

Intervention	Mean	Standard deviation
No education module before completing simulation (n=42)	0.627	0.127
Education module before completing simulation (n=42)	0.685	0.136

Research Question 2

To answer this research question, the average of all attempt 1s on all three days was compared to the average of attempt 2s on all three days. Analyses included descriptive statistics of the number of groups who attempted to obtain consent at each time point, means and standard deviations for each day’s average of attempt 1s and attempt 2s, and an independent t-test to compare outcomes scores of these conditions for each day (Table 2).

**Table 2:** The means between attempt 1 and attempt 2 on each of the simulation days as a method of analyzing the impact of deliberate practice.

	Attempt 1			Attempt 2		
Day	n	Mean	Standard deviation	n	Mean	Standard deviation
1	13	0.581	0.129	17	0.667	0.088*
2	12	0.620	0.160	15	0.704	0.124
3	10	0.722	0.108	17	0.648	0.158

On day 1, there was a significant difference between groups’ scores on attempt 1 and attempt 2, *t*(28) = 2.154, p = 0.02 with a medium effect size, d = 0.794. This shows that deliberate practice alone resulted in increases in consent scores. On day 2, there was a non-significant increase between average groups’ scores on attempt 1 and attempt 2, *t*(25) = 1.526, p = 0.070, with a medium effect size, d = 0.591. On day 3, there was a non-significant decrease between average groups’ scores on attempt 1 and attempt 2, *t*(25) = 1.326, p = 0.098, with a medium effect size, d = 0.528.

It is notable that on day 3, there were nearly twice as many tries for attempt 2 than for attempt 1. In comparing the group scores on attempt 2 for those who tried both attempt 1 and attempt 2 to just those who tried attempt 2, the groups who did both attempts scored better on attempt 2 (mean = 0.704) than those who just tried attempt 2 (mean = 0.648), although this difference was not statistically different, *t*(13) = 0.788, p = 0.222, with a small effect size, d = 0.415.

Research Question 3

To answer this research question, we compared the groups who did attempt 2 on day 2 (those who practiced one and then received the intervention) to those who did attempt 1 on day 3 (those who received the intervention without practice) (Table 3).

**Table 3:** Mean values between attempt 2 day 2 and attempt 1 day 3 for deliberate practice model as a method of comparing deliberate practice to study intervention.

Day	Attempt	Number of groups who attempted	Mean	Standard deviation
Day 2	Attempt 2	15	7.04	0.124
Day 3	Attempt 1	10	0.722	0.108

In comparing scores of students on attempt 2 on day 2 to those on attempt 1 on day 3, there is no statistically significant difference in scores *t*(23) = 0.385, p = 0.352, with a small effect size, d = 0.157. This indicates that the students who had deliberate practice and the intervention had the same scores as those who just received the intervention.

## Discussion

Results of our study suggest that our intervention of incorporating a structured didactic pamphlet outlining the elements of obtaining parental consent over a telephone encounter for pediatric patients was successful, leading to an overall better outcome of communication as measured by the rubric. Our data also suggest that the deliberate practice had equivalent benefit to communication outcomes as the intervention. This aligns with previous work demonstrating the efficacy of deliberate practice in gaining such skills [12]. While the research team of this study would have hypothesized that the intervention would have had more significant benefit, this result shows that either deliberate practice or the intervention would be beneficial. Essentially, this study suggests that bypassing deliberate practice by just providing the intervention results in the same outcomes and that there is no added benefit of practicing both deliberate practice and introducing the intervention. Of note, due to the limitations of this study, there was a low number of groups that had both deliberate practice and the intervention, which influenced the lack of statistical significance between deliberate practice and our intervention. As previously outlined, there exists a lack of formal training on obtaining informed consent despite the relevance of the skill noted in previous literature [2,3]. The implications of this study’s results suggest that incorporating the intervention of this study within the curriculum of graduate medical education can help bridge this knowledge gap.

Additionally, in looking at the raw data, it was apparent that the issue of legal guardianship was unsuccessfully approached by every single group that obtained parental consent throughout the simulation. The authors suspect this reflects the course and/or program curriculum rather than an influence of the simulation scenarios or the structure of the study. Despite the pamphlet broaching this subject, students did not confirm the medical-legal decision-making capacity of their parents. This is an important topic to place greater focus on in the pediatric medicine teaching modules of the course/program curriculum in the future. Previous literature has shown that many medical students do not feel competent or confident in matters of obtaining parental consent, which include legal guardianship [8]. These results confirm that this study cohort may have felt similarly and therefore universally failed to address the issue of legal guardianship. Further emphasis on these matters may help students feel more confident regarding these matters.

All in all, the data support that there is a significant improvement in the student’s ability to obtain telephone parental consent for pediatric patients after receiving the intervention, and this is the largest improvement. There is still improvement between performance in the first simulation and the second simulation, although it is not as great as the improvement following the intervention. This is expected as the deliberate practice model enables students to debrief and learn from the first simulation and improve the second time, regardless of whether they have been given additional training in the interim. The data also shows no clear improvement when the intervention and deliberate practice combined. This is likely a result that each leads to powerful results on its own, but more studies are necessary to confirm this. It is noteworthy that deliberate practice delivers such positive results as this is what the simulation literature suggests we should expect from this model.

Examining limitations of the study, the authors were not able to identify specifically which student was taking the initiative of obtaining consent each time. As a result, it was unclear if a single student was obtaining consent for the group in both the first and second case, or if different students from the same group were performing this action. This makes it difficult to interpret the data when comparing performance even in the same group from one simulation to the next. There were also confounding variables of multiple students in each group, as well as not all patient cases were attempted by each group each time. This could result in the appearance that a group consented fewer patients when, in fact, they simply encountered fewer patients.

Another limitation was the relatively small number of groups overall who completed both the deliberate practice and who received the intervention. As a result, we were unable to detect statistical significance between the intervention and the effect of deliberate practice. Also, there was no method for tracking, if obtaining consent was even attempted. In other words, the student may not have even called the parent and just treated the minor without attempting to obtain consent.

Lastly, we must consider if the emergency department setting of the simulations impacted the students’ decisions to obtain and/or their performance in obtaining parental consent. If the simulation were a primary care scenario that was less intense, the students may have performed differently.

Due to these limitations, it is not clear if the data are a result of limitations in the didactic, in the simulation itself, or student understanding, particularly when addressing the issue of emphasis of legal guardianship verification. All these limitations help inform possible future directions of research. The authors are eager to gather additional data with a larger number of groups as well as implementing improved tracking of which individual is performing each action. Specifically, gathering data on which minor patients are treated without any attempt at obtaining consent, and tracking which student is making the phone call to the parent to obtain consent. This will help determine if improvement or lack of improvement between cases, as a result of the intervention or deliberate practice, versus varying performance between different students.

Future studies could also include a survey of students or post-simulation focus groups to obtain additional quantitative and qualitative data. The study could be conducted using primary care cases, not in an emergency department, to look at the impact of clinical setting on student performance. It would also be informative to study how students perform obtaining consent, face-to-face, rather than over the phone. Being able to interact and use, and observe nonverbal communication may impact their performance. The authors would also like to research the performance of prehospital healthcare providers in the same realm of obtaining consent.

## Conclusions

This study supports the conclusion that there is a significant benefit to adding structured didactic information on obtaining parental consent for pediatric cases for medical learners. Both the intervention and deliberate practice models have benefits in increasing positive communication outcomes for students, which can help alleviate the anxiety and lack of preparation that many providers face in their pediatric practices. This study can be improved in future studies by increasing the number of groups studied, as well as by improved tracking of which student is responsible for obtaining each consent, to showcase the efficacy of the intervention and deliberate practice further.

## Figures and Tables

**Figure 1:**
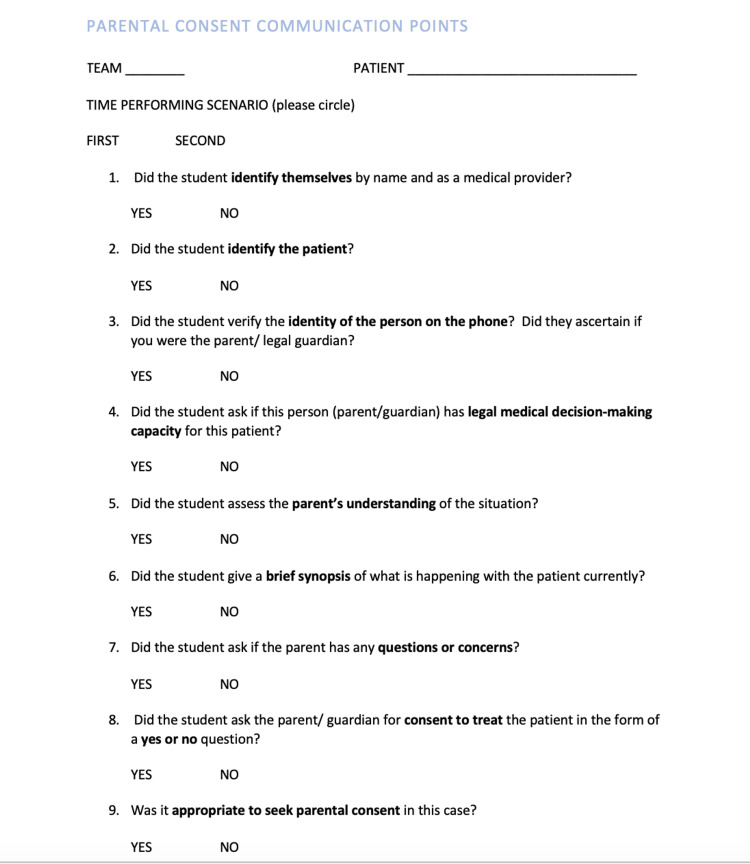
Communication outcomes grading rubric. Rubric was used to assess communication outcomes that were taught in the intervention.
